# Supplementary material for: Fecal Microbiome Features Associated with Extended-Spectrum β-Lactamase-Producing Enterobacterales Carriage in Dairy Heifers
Source: Animals (Basel). 2022 Jul 6;12(14):1738. doi: 10.3390/ani12141738 (PMC9311658; doi:10.3390/ani12141738)
Supplement: Supplementary file 1 [file animals-12-01738-s001.zip › animals-170339-supplementary.pdf]

Table S1: Results of farm demographic survey, including proportion of samples with detectable ESBL-producing *Enterobacterales* (ESBL-PE).

Farm	N cows	N milking cows	Dogs (Yes/No	Cats (Yes/No)	Mastitis %	Mortality %	Morbidity %	Density (meter/ cow)	Bedding	Colostrum	Milk feeding	Age of weaning (days)	Cooling system	Antibiotic administration (Yes/No)	Cleaning method	N Cows sampled	Number of ESBL+ samples	E.coli-ESBL	Prevalence of E.coli ESBL
CH	700	370	No	No	0.300	0.060	0.030	9	Oil shale	Individual	Milk replacement	29	Both	No	Tractor	20	2	2	10.0
GAN	550	305	Yes	Yes	0.180	0.018	0.050	10	None	Pooled	Milk replacement	63	Nebulizer	Yes	Automatic shovel	20	2	2	10.0
GI	590	230	Yes	Yes	0.130	0.020	0.060	9	Oil shale	Individual	Whole milk	60	Both	No	Tractor	20	2	0	0.0
NB	370	180	Yes	No	0.300	0.100	0.070	10	Oil shale	Individual	Milk replacement	60	Both	No	Automatic shovel	20	3	3	15.0
NS	614	320	Yes	Yes	0.300	0.066	0.086	10	None	Pooled	Both	70	Fan	No	Both	18	8	6	33.3
R	513	260	Yes	Yes	na	0.020	na	11	None	Pooled	Milk replacement	40	Both	No	Automatic shovel	20	10	9	45.0
SI	634	262	Yes	No	0.100	0.040	0.010	12	Oil shale	Pooled	Both	60	Both	Yes	Tractor	20	20	20	100.0
V	290	190	Yes	No	0.175	0.010	0.030	11	Straw	Pooled	Whole milk	60	Fan	No	Tractor	19	8	6	31.6
